# Investigations on Oxidation Behavior of Free-Standing CoNiCrAlYHf Coating with Different Surface Roughness at 1050 °C

**DOI:** 10.3390/ma16124282

**Published:** 2023-06-09

**Authors:** Nadimullah Hakimi, Peng Song, Tabasum Huma, Dadallah Hanifi, Danish Bakhshyar, Wahab Abdul Ghafar, Taihong Huang

**Affiliations:** 1 Faculty of Materials Science and Engineering, Kunming University of Science and Technology, Kunming 650093, China; nadim.hakimi123@gmail.com (N.H.); tabassumhumaanwar@gmail.com (T.H.); dadallah2021@gmail.com (D.H.); 2 Faculty of Civil Aviation and Aeronautics, Kunming University of Science and Technology, Kunming 650093, China; 3 Faculty of Chemical Engineering, Kunming University of Science and Technology, Kunming 650093, China; danish7444@yahoo.com; 4 Faculty of Civil Engineering and Mechanics, Kunming University of Science and Technology, Kunming 650500, China; ghafarw54@gmail.com; 5 Yunnan Engineering Research Center of Metallic Powder Materials, Kunming 650093, China

**Keywords:** free-standing CoNiCrAlYHf coating, surface roughness, alumina scale, oxidation kinetics

## Abstract

MCrAlYHf bond coats are employed in jet and aircraft engines, stationary gas turbines, and power plants, which require strong resistance to oxidation at high temperatures. This study investigated the oxidation behavior of a free-standing CoNiCrAlYHf coating with varying surface roughness. The surface roughness was analyzed using a contact profilometer and SEM. Oxidation tests were conducted in an air furnace at 1050 °C to examine the oxidation kinetics. X-ray diffraction, focused ion beam, scanning electron microscopy, and scanning transmission electron microscopy were employed to characterize the surface oxides. The results show that the sample with Ra = 0.130 µm demonstrates better oxidation resistance compared to Ra = 7.572 µm and other surfaces with higher roughness in this study. Reducing surface roughness led to a decrease in the thickness of oxide scales, while the smoothest surface exhibited increased growth of internal HfO_2_. The β-phase on the surface with Ra = 130 µm demonstrated faster growth of Al_2_O_3_ compared to the γ-phase. An empirical model was suggested to explain the impact of surface roughness on oxidation behavior based on the correlation between the surface roughness level and oxidation rates.

## 1. Introduction

Materials used to produce components for high-temperature equipment, such as aircraft engines, gas turbines in jet motors, stationary turbines, and power plants, must have several exceptional features, such as low-temperature ductility, high creep resistance, and oxidation resistance at a wide range of working temperatures, environments, and pressure conditions [1,2,3,4]. The Ni-based superalloys family meets these specifications [5]. The durability of metals decreases at high temperatures owing to high-temperature corrosion, which is primarily caused by the interaction between metals and oxygen [6]. Turbine blades and other high-temperature components of aerojet engines have greatly benefited from the use of Ni-based single-crystal (SC) superalloys because of their superior high-temperature mechanical capabilities [7]. Oxidation is one of the most critical evaluation indicators of the high-temperature performance of high-temperature alloy systems because it can cause de-alloying corrosion, loss of surface strength, cracking, and eventual collapse [8]. Thermal barrier coatings are often used to prevent the oxidation of alloys [9,10]. Significant thermal disturbances, mechanical strain, and partial stress loading on turbine blades during operation may result in the cracking or spalling of the protective coating [11]. However, coatings cannot be applied to certain locations, such as film perforations on cooling turbine blades [12]. Consequently, understanding the substrate behavior in an oxidative environment is essential [13]. However, the morphological compositions and structures of the oxide scales of solitary Ni-based crystal superalloys are complex [14]. Currently, the major determinant of oxidation behavior is the composition of various elements, including Al, Ta, Si, Hf, and Re [15,16]. Numerous studies have investigated the oxidation behavior of Ni-based superalloys [17], including their oxidation kinetics and the oxide layer microstructure [18,19]. Owing to the feasibility of the experiment, the oxidation kinetics are widely described in terms of changes in mass [20]. Generally, the mass gain is speculated to follow a curved response to the oxidation duration. However, recent studies have reported several phases of parabolic curves [21,22].

Pei et al. [23] differentiated four oxidation phases in the calculated parabolic curves for the Ni–4.0Cr–5.7Al single crystal superalloy. Pfennig and Fedelich [24] observed two distinct oxidation phases on a single crystal PWA1483. Another study [25] suggested two-step oxidation kinetics. Although various studies have identified several stages of the oxidation kinetics, the differences between a single parabolic law and numerous phases have not been fully acknowledged. Extensive research [26,27,28] has confirmed that surface roughness may change the oxide microstructure of Ni-based superalloys, thereby influencing their oxidation resistance [29]. Evans [4] and Sun et al. [30] demonstrated that increasing the initial surface roughness resulted in a decreasing oxide resistance. Multiscale characterization approaches were used to investigate the oxide-scale microstructure and composition generated on a Ni–Co-based superalloy following oxidation at 900 °C in air for 100 h. Continuous surface oxide scale and interior oxides were preferentially formed along the grain boundaries. Transmission electron microscopy (TEM) and transmission Kikuchi diffraction (TKD) indicated that the grain boundary oxides were mostly equiaxed nanosized Al_2_Ti_7_O_15_. Al_2_Ti_7_O_15_ has several plane defects that can act as rapid diffusion routes for the inward diffusion of oxygen, causing preferential grain boundary oxidation [7]. An Ni-based superalloy with an ultrafine-grained surface (UFGS–GTD111) and its polycrystalline equivalent (CP–GTD111) were compared for oxidation in Ar + 20% O_2_ at 900 °C. The surface of UFGS–GTD111 exhibited more GBs and dislocations than that of CP–GTD111, which accelerated the diffusion of scale-forming elements and oxygen anions near the oxidation zone. UFGS–GTD111 rapidly formed a three-layer oxide film (outer Ti-, middle Cr-, and inner Al-rich), which improved oxidation. Grain refinement lowered the Al concentration required to form an essentially continuous alumina layer [31,32].

Herein, the effects of surface roughness were investigated on the oxidation behavior of a free-standing CoNiCrAlYHf coating at 1050 °C in the presence of dry air. The surface roughness was evaluated using a contact profilometer and scanning electron microscope (SEM). Energy-dispersive spectroscopy (EDS) was used to determine the composition of the oxidized material, and SEM was used to identify the structure of the material and its ability to remain intact. TEM was used to investigate the development in the Al_2_O_3_ scale and its microstructure at 1050 °C. In addition, the positions of the active elements (Hf) in the oxide scales were analyzed. The results show that the sample with Ra = 0.130 µm demonstrates better oxidation resistance compared to Ra = 7.572 µm and other surfaces with higher roughness in this study. Reducing surface roughness led to a decrease in the thickness of oxide scales, while the smoothest surface exhibited increased growth of internal HfO_2_. At present, no reports have described the growth characteristics, microstructure, or composition of oxide scales formed on a free-standing CoNiCrAlYHf coating with different surface roughness at high temperatures in dry air. Therefore, we decided to conduct this investigation.

## 2. Material and Methods

Herein, a commercially available free-standing CoNiCrAlYHf was investigated. The chemical composition is listed in Table 1.

**Figure 1:**
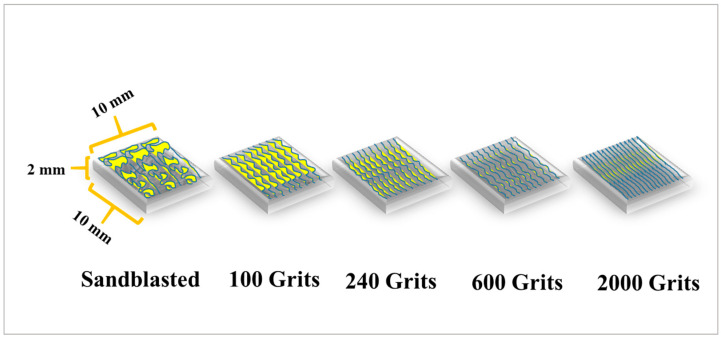
Schematic of the free-standing CoNiCrAlYHf coating. Square-shaped samples with measurements of 10 mm × 10 mm × 2 mm were machined from the block and polished to different surface roughness.

Square-shaped specimens with dimensions of 10 mm × 10 mm × 2 mm were machined from cast free-standing CoNiCrAlYHf blocks. Subsequently, all specimens were polished using a sandpaper and a polish machine up to 2000 grits (100–240, 400, 600, 800, 1000, 1200, 1500, and 2000 grits). All specimens were subjected to the same surface preparation. Next, five samples were selected as representative samples, which were mechanically polished to different surface roughness using abrasive paper, except for one sample for which a sandblasting machine was used in the workshop. The surface of sample 1 was sandblasted using a sandblasting device, resulting in a rough surface; the surface of sample 2 was polished up to 100 grits using a sandpaper; the surfaces of sample 3 and 4 were ground up to 240 and 600 grits, respectively, using abrasives to produce different rough surfaces; and the surface of sample 5 was polished up to 2000 grits using a sandpaper, resulting in a smooth surface (Figure 1).

**Figure 2:**
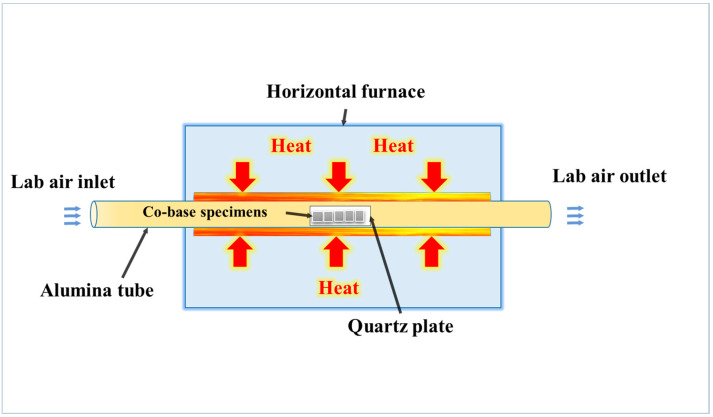
Schematic of the oxidation test device.

Subsequently, the samples were ultrasonically cleaned in ethanol and dried using a blower. The initial surface roughness was estimated using SEM images, and ImageJ software was used to build 3D surface images. A standard contact profilometer (SJ-210, Mitutoyo, Aurora, IL, USA) was used to quantify the surface roughness. The sample surfaces were examined using SEM before the oxidation test to identify any possible scratches. The initial weights of all the samples were then recorded before they were cleaned again and subjected to the air oxidation test. This test was conducted in a horizontal furnace for 216 h at 1050 °C. A schematic of the oxidation device is shown in (Figure 2).

Digital laboratory weight scales were used to determine the actual weight of each sample at the beginning of the oxidation tests. After every 24 h of oxidation, specimens were taken out of the furnace, cooled for 30–40 min at room temperature (~24 °C), weighed again, and returned to the furnace. The mass differences were documented during the oxidation test at 1050 °C for samples with different surface roughness. Throughout the oxidation test, the weight of each specimen with various surface roughness values was recorded, and the mass change was accurately calculated. X-ray diffraction (XRD) analysis was performed to identify the type of oxidation that occurred in the materials and the corresponding phase composition of the samples. Optical morphologies were used to observe the differences in surface scratches and forms before and after the oxidation test. Surface SEM was used before and after the oxidation test to monitor the surfaces of the specimens in close detail. Standard operating procedures were followed to prepare the metallographic cross-sections. SEM was used to observe and investigate the cross-sections. TEM was employed to investigate the locations of the active elements (Al and Hf) on the smoothest surface (2000-grit samples) in the oxide scale at 1050 °C in air and the growth and microstructure of the Al_2_O_3_ scale.

## 3. Results

### 3.1. Surface Roughness

**Figure 3:**
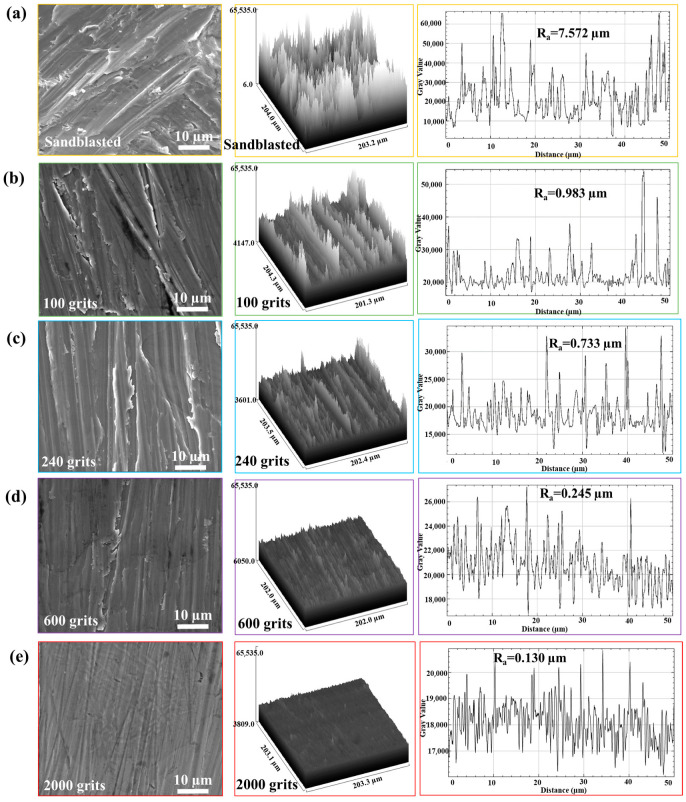
Three-dimensional surface pictures created using scanning electron microscopic images and ImageJ2 software. Typical contact profilometer (SJ-210 Mitutoyo) measurements of surface roughness. (**a**) Sandblasted surface, (**b**) 100 grits, (**c**) 240 grits, (**d**) 600 grits, and (**e**) 2000 grits.

Representative samples were selected from each surface preparation procedure, and the corresponding surface roughness values were recorded. The surface preparation conditions were analyzed using SEM. The SEM images and ImageJ2 software (Fiji GPL v3) were used to generate 3D surface images (Figure 3). Typical contact profilometer measurements of the surface roughness were performed using an SJ-210 Mitutoyo. The surface roughness (Ra) measurements of the sandblasted (Figure 3a), 100-grit (Figure 3b), 240-grit (Figure 3c), 600-grit (Figure 3d), and 2000-grit samples (Figure 3e) are Ra = 7.572, 0.983, 0.733, 0.245, and 0.130 µm, respectively.

**Figure 4:**
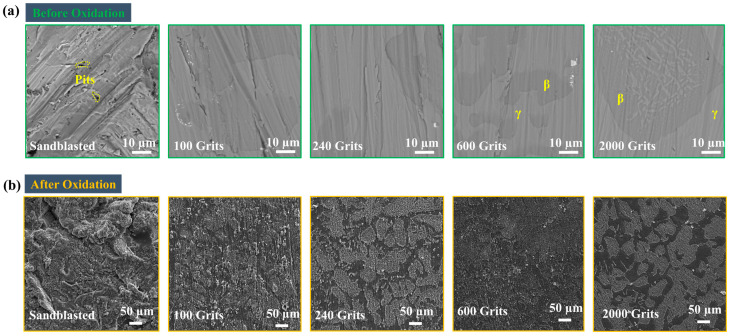
Scanning electron microscopic images of the free-standing CoNiCrAlYHf coating surfaces before and after the oxidation test. (**a**) Surface SEM before oxidation, (**b**) Surface SEM after oxidation.

Thus, the sandblasted surface and 2000-grit samples exhibit the highest and lowest roughness level, respectively. Figure 4a demonstrates the SEM images of the free-standing CoNiCrAlYHf coating surfaces before the oxidation test. The surface SEM was used to ensure that SiC particles were not entrenched at the top of the surfaces in any of the investigated samples. The micromorphologies of the surfaces of the samples with different roughness levels were observed, which were produced by polishing them with abrasive paper using a polishing machine, followed by mechanical polish using an abrasive paper. A sandblasted sample was polished with a polishing machine, and its surface was sandblasted using a sandblasting machine, which created pits on the surface and made it rough and bumpy. A 100-grit sample was mechanically polished from top to bottom in the scratch direction (Figure 4a).

### 3.2. Isothermal Oxidation Test at 1050 °C

**Figure 5:**
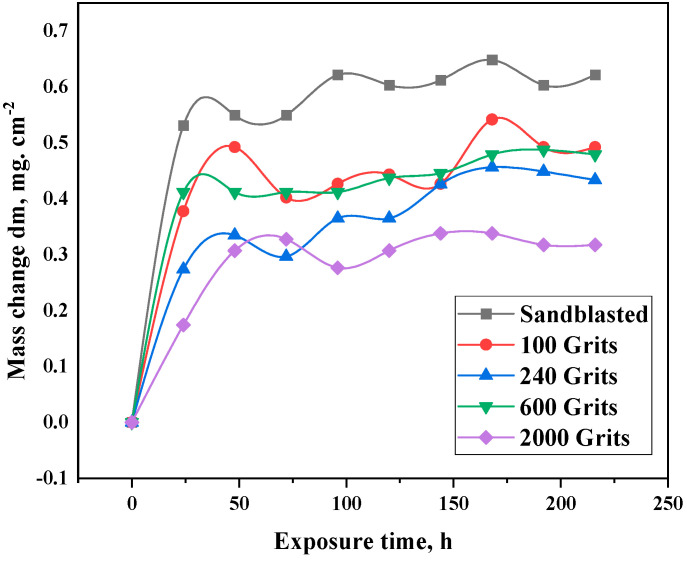
Mass-change graph of free-standing CoNiCrAlYHf coating samples after oxidation at 1050 °C for 216 h.

The isothermal oxidation test was carried out at 1050 °C to investigate the effect of varying surface roughness on the free-standing CoNiCrAlYHf coating oxidation behavior. The alloy with a sandblasted sample exhibits a mass change nearly twice that of the alloy with a polished surface of 2000 grit (Figure 5). A considerable difference in the mass change is observed after 20 h of exposure. This trend was evident at the end of the isothermal oxidation test. An increase in mass is observed during the first 20 h, and then the mass gain stabilizes at a nearly constant value. A small variation is observed at 168 h, which is the most significant value for all specimens. The sandblasted, 100-grit, 240-grit, 600-grit, and 2000-grit samples exhibit a mass change per unit area of approximately 0.6, 0.5, 0.39, 0.4, and 0.3 mg·cm^−2^, respectively (Figure 5). Optical morphology results after the oxidation test reveal that the superalloy changes color during oxidation at 1050 °C, owing to the formation of an oxide layer on the top of the surface. The color of the oxide layer is blue, which is more noticeable on surfaces that have been sandblasted, with a surface roughness of Ra = 7.572 µm, and the color of the sample with Ra = 0.130 µm changes to a blueish gray.

### 3.3. Oxide Phase Composition after 216 h of Oxidation

**Figure 6:**
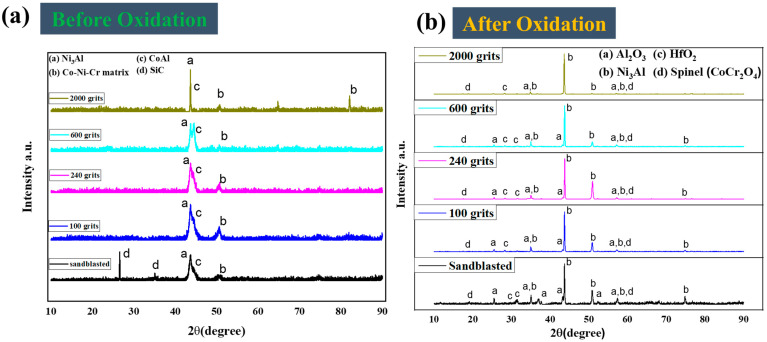
X-ray diffraction patterns of the free-standing CoNiCrAlYHf coating surfaces before and after the oxidation test at 1050 °C with various levels of surface roughness. (**a**) XRD before oxidation, (**b**) XRD after oxidation.

The XRD semi-quantitative analysis technique was used before and after an oxidation test on free-standing CoNiCrAlYHf to determine the oxide phases present on the surface of the samples during 216 h of oxidation at 1050 °C in air. Figure 6a depicts the XRD patterns of the sandblasted, 100-grit, 240-grit, 600-grit, and 2000-grit specimen surfaces before the oxidation test. Ni_3_Al, CoAl, and Co–Ni–Cr matrices are identified on the surfaces of the specimens. Moreover, SiC is observed on the sandblasted surface, which is attributed to the sand entering the surface during sandblasting. Figure 6b shows the XRD patterns of oxidized specimen surfaces (sandblasted, 100-grit, 240-grit, 600-grit, and 2000-grit) after 216 h of oxidation at 1050 °C in air. The oxides of Ni_3_Al, Al_2_O_3_, HfO_2_, and spinel (CoCr_2_O_4_) are observed on the surfaces. The intensities of the Ni_3_Al and Al_2_O_3_ diffraction peaks on the sandblasted, 100-grit, and 240-grit surfaces are more significant than those on the 600- and 2000-grit surfaces. In addition, when the HfO_2_ and spinel are low in quantity with smoother surfaces, the intensities of the diffraction peaks decrease. The intensities of the diffraction peaks of Ni_3_Al, Al_2_O_3_, HfO_2_, and spinel (CoCr_2_O_4_) of different surface roughness follow the order: sandblasted surface > 100-grit sample > 240-grit sample > 600-grit sample > 2000-grit sample, implying that the smoother the polishing surface, the thinner the oxide scale becomes after 216 h of oxidation in air at 1050 °C.

### 3.4. Surface Microstructural and Cross-Section Analyses

**Figure 7:**
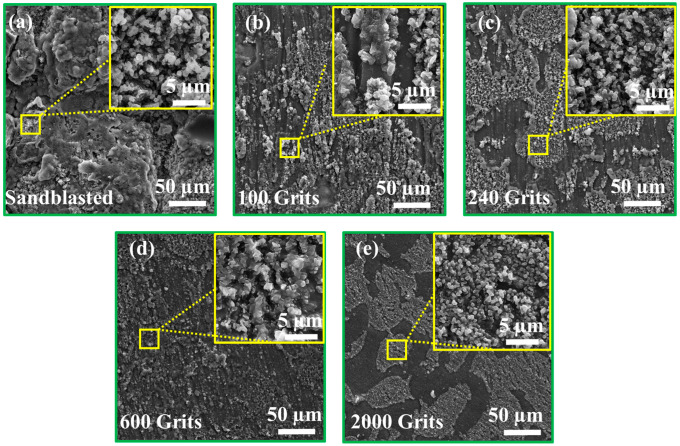
Scanning electron microscopic images of the free-standing CoNiCrAlYHf coating surfaces after the oxidation test: (**a**) sandblasted, (**b**) 100-grit, (**c**) 240-grit, (**d**) 600-grit, and (**e**) 2000-grit surfaces.

SEM analysis was performed to characterize the oxides surfaces after 216 h of exposure to a high temperature. The roughness levels of each of the specimens are shown in Figure 4b, illustrating the differences in the oxidation behavior of surface of each sample. The surface of each specimen is covered with a fresh layer of oxide particles. All the five sample types exhibit distinct surface characteristics. However, the oxide particles on the sandblasted surface exhibit larger diameters than those on the samples with a surface roughness of 100 and 240 grits (Figure 7), indicating a gradual increase of the oxides and a uniform oxide layer. The oxide particles in the 600-grit sample are widely separated from each other, which coincides with the oxidation behavior of the sample, shown in Figure 5. The 2000-grit sample exhibits the smoothest surface; the Ni_3_Al and HfO_2_ are speculated to form the oxide layer in a given surface area. This increased diffusion helps rapidly nucleate the oxide particles, leading to their higher production. This illustrates the SEM image at various magnifications. An oxide particle layer is evident on the surface of each sample. At high magnification, the surfaces of all five types of samples exhibit comparable characteristics. In addition, the oxide particles on the surface of the sandblasted sample are larger than those on the surfaces of the 100, 240, 600, and 2000-grit samples.

**Figure 8:**
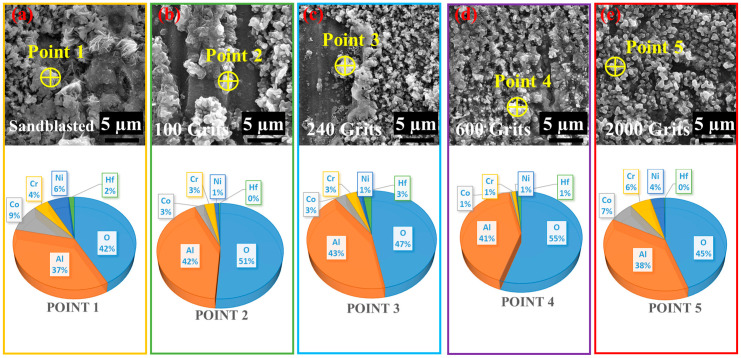
Surface morphologies and energy-dispersive spectroscopic analyses of the free-standing CoNiCrAlYHf coating surfaces after oxidation at 1050 °C for 216 h with different surface roughness: (**a**) sandblasted, (**b**) 100-grit, (**c**) 240-grit, (**d**) 600-grit, and (**e**) 2000-grit surfaces.

The energy distribution of the oxygen scales on the surface was detected using EDS analysis (Figure 8). The molecular makeup of all samples and selected points under extremely high magnification are summarized in Figure 8a–e. The oxide scales with the sandblasted, 100-grit, 240-grit, 600-grit, and 2000-grit surfaces depict mass percentages of 42%O–37%Al–9%Co–4%Cr–6%Ni–2%Hf, 51%O–42%Al–3%Co–3%Cr–1%Ni–0%Hf, 47%O–43%Al–3%Co–3%Cr–1%Ni–3%Hf, 55%O–41%Al–1%Co–1%Cr–1%Ni–1%Hf, and 45%O–38%Al–7%Co–6%Cr–4%Ni–0%Hf, respectively. The compositions of the two elements Al and O are comparable in all samples with a minor fraction of Co, Cr, and Ni elements. Furthermore, the Hf concentration in the specimens with the sandblasted and 240-grit surfaces is higher, whereas Co and Ni elements are observed in relatively higher fractions on the sandblasted and 2000-grit surface (Figure 8).

**Figure 9:**
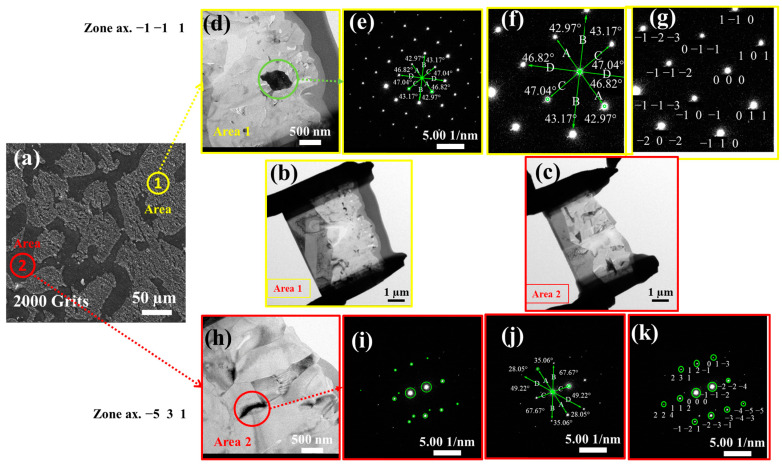
(**a**) SEM image of an oxidized 2000 grit surface after 216 h oxidation test at 1050 °C; (**b**) Cut portion of the surface made by FIB on Area 1, demonstrating the region’s significant oxide growth on the surface; (**c**) Cut portion of the surface made by FIB on Area 2, demonstrating the region with no oxide developments on the surface; (**d**–**g**) TEM image of the interior layer of the oxide scale developed on a 2000 grits surface on Area one; (**h**–**k**) TEM image of the interior layer of the oxide scale on a 2000 grits surface on Area two.

Figure 9a demonstrates the SEM image of an oxidized 2000-grit surface after 216 h of oxidation at 1050 °C in a dry air environment with two distinct areas: one with considerable oxide growth (Area 1) and the other with no oxide growth on the surface (Area 2). TEM was performed on the specimens with increased spatial resolution. A specimen that is sufficiently thin to transmit an electron beam is necessary, which is possible using a focused ion beam (FIB). FIB was used to make surface cuts in Area 1 and 2 (Figure 9b,c). TEM was used to investigate the interior and external structures of the oxide layers. Figure 9d demonstrates TEM images of the exterior and interior layers of the oxide film formed in Area 1, indicating the significant oxide growth on the surface. In contrast, Figure 9h shows the TEM images of the exterior and interior layers of the oxide film formed in Area 2, where no significant oxide growth is observed on the surface. Figure 9e–g,i–k demonstrate the matching patterns of the selective area electron diffraction (SAED).

**Figure 10:**
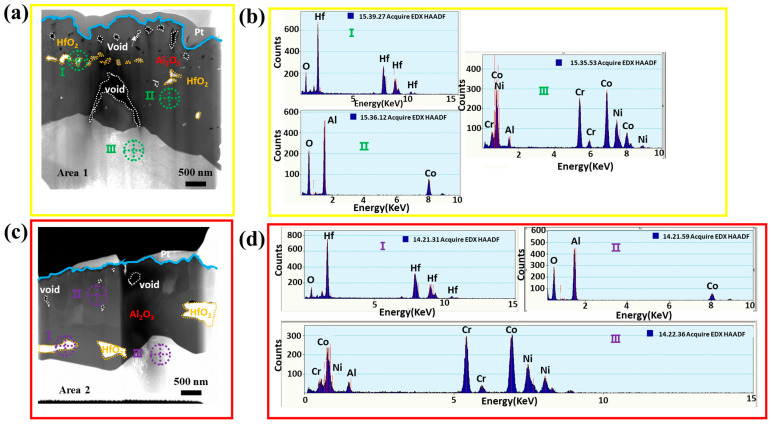
STEM cross-sectional images of the oxide scale formed on free-standing CoNiCrAlYHf coating with the surface roughness of 2000 Grits (Ra = 0.130 µm) after oxidation at 1050 °C for 216 h in two different areas of surface: (**a**,**b**) Area 1, (**c**,**d**) Area 2 (Cut by FIB).

Figure 10 shows the scanning transmission electron microscopy (STEM) cross-sectional images and EDS spectra of the oxide scale produced on the free-standing CoNiCrAlYHf coating after 216 h of oxidation in dry air on a sample with a surface roughness Ra of 0.130 µm after being polished with 2000-grit sandpaper. Both Area 1 and 2 surfaces exhibit a characteristic two-layer alumina grain structure (Figure 10a–d). Both areas include thick oxide scales that virtually reveal Al_2_O_3_ and a layer of shattered HfO_2_ when subjected to EDS analysis. The thick oxide scales in Area 1 exhibit significant oxide growth on the surface, fast growth of Al_2_O_3_ in the internal and external oxide layers, and a thin, straight layer of HfO_2_ that causes numerous voids in the oxide scales (Figure 10a,b). In contrast, the oxide scale in Area 2 is thin, dense, and has fewer voids. Compressed layers of Al_2_O_3_ and dispersed grains of HfO_2_ are observed in the bottom layer of the oxide scale, similar to the substrate (Figure 10c,d).

**Figure 11:**
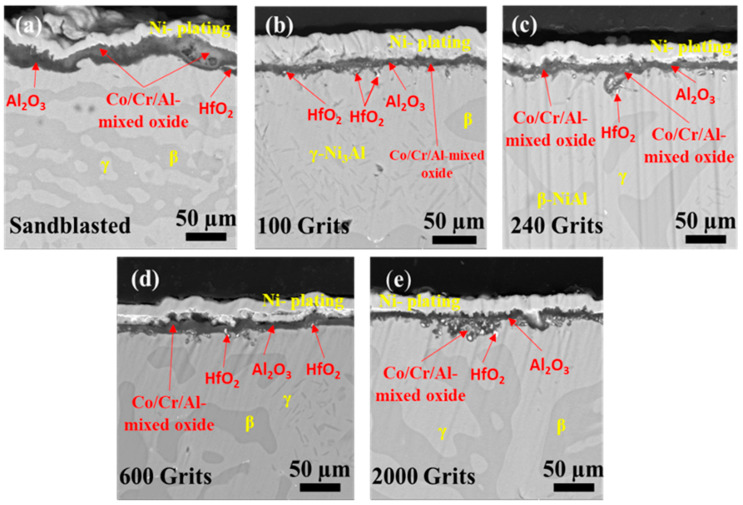
Cross-sectional SEM images of free-standing CoNiCrAlYHf coating after oxidation at 1050 °C for 216 h with different surface roughness (**a**) Sandblasted surface, (**b**) 100 grits, (**c**) 240 grits, (**d**) 600 grits, and (**e**) 2000 grits.

Figure 11a–e demonstrate the cross-sectional images of samples with different surface roughness obtained after the isothermal oxidation test. The phases of the alloy, such as γ-Ni_3_Al and β-NiAl with low and high Al content, respectively, are readily seen [33]. The sample with a sandblasted surface is characterized by Al_2_O_3_ and Co-/Cr-/Al-rich oxide production (the most likely mixture of spinel (CoCr_2_O_4_) with the local presence of Al_2_O_3_ and HfO_2_ oxides) (Figure 11a). Furthermore, this surface exhibits the thickest oxide scale and the lowest amount of internal oxide. Some pores are visible on the exterior oxide scale. The oxide scale produced on the sample with a 100-grit surface roughness (Figure 11b) is lighter and thinner than that on the sandblasted sample. In addition, the formation of Al_2_O_3_ and HfO_2_ at the oxide-scale alloy interface is observed. However, HfO_2_ precipitates are localized beneath the exterior oxide scale and appear less firmly incorporated into the alloy grain boundaries than in the case of the sandblasted surface. The internal oxides near the surface resemble a split, and the HfO_2_ concentrations are higher in that area. The internal oxide scale developed on the 240-grit surface roughness sample is scattered near the surface, with HfO_2_ making up most of the scattered internal oxide (Figure 11c). In addition, the oxide scales of Al_2_O_3_ and Co-/Cr-/Al-mixed oxides are formed on the exterior oxide of the alloy. The formation of a Co-/Cr-/Al-mixed oxide, Al_2_O_3_, and HfO_2_ in the oxide scale, as well as many pores and fissures in the exterior oxides, are observed on a sample with a surface roughness of 600 grit (Figure 11d). The cross-sectional image of the sample with a 2000-grit surface roughness (Figure 11e) demonstrates a significantly thinner oxide scale than those formed on the other samples. The compact oxide scale primarily comprises Al_2_O_3_ and HfO_2_ oxides, with a considerable inner oxide scale located beneath the HfO_2_.

## 4. Discussion

Variations in the surface roughness were produced using various surface treatment techniques, which caused a substantial impact on the oxidation behavior of each investigated alloy. Furthermore, the observed impact has an effect on short-term as well as long-term isothermal air oxidation of the examined alloys. This is demonstrated by the presence of the observed effect. Examination of the free-standing CoNiCrAlYHf coating with a more recognizable chemical makeup revealed that this alloy exhibited the effects of surface treatment on the oxidation behavior. Three elements were identified based on the preliminary analysis of the free-standing CoNiCrAlYHf coating, which were speculated to be primarily responsible for the observed phenomena. The ratios of roughness to surface development, environment, method of preparation, and introduced defects are examples of these variables. An increase in the surface roughness was observed based on the surface development ratio.

### Effects of Surface Roughness on the Oxidation Behavior of Free-Standing CoNiCrAlYHf Oxide Scale

**Figure 12:**
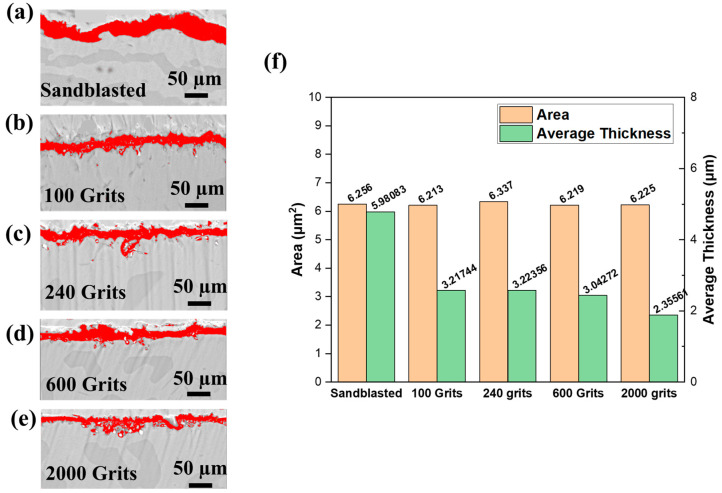
The average thickness and area of the oxide scale formed on the free-standing CoNiCrAlYHf coating after being oxidized at 1050 °C for 216 h with different surface roughness in air (**a**) Sandblasted surface with Ra = 7.572 µm; (**b**) 100 grits surface with Ra = 0.983 µm; (**c**) 240 grits surface with Ra = 0.733 µm; (**d**) 600 grits surface with Ra = 0.245 µm; (**e**) 2000 grits surface with Ra = 0.130 µm; and (**f**) a histogram of the results.

To investigate the effects of different surface roughness on the oxidation behavior of the free-standing CoNiCrAlYHf coating and initial growth of the oxide scale, the characterization of the scale area and average thickness on the cross-section of the investigated alloys are shown in Figure 12. The scale area and average thickness formed in the oxidation test are significantly larger in the sandblasted sample, with surface roughness Ra = 7.572 µm at 1050 °C in dry air for 216 h compared to that produced in the 2000-grit surface with Ra = 0.130 µm.

The average thickness of oxide scales for 100-grit (Ra = 0.983 µm) and 240-grit (Ra = 0.733 µm) surfaces are 3.21744 and 3.22356 µm, respectively, which are nearly identical. A small discrepancy is attributed to the action of some strip of interior oxide. The average thickness of the oxide scale is 3.04272 µm for a 600-grit surface with Ra = 0.245 µm; hence, a low surface roughness reduces the oxidation resistance of the free-standing CoNiCrAlYHf material, whereas a high surface roughness increases the oxidation resistance. The environment exhibited high oxidation resistance. Furthermore, the area and average thickness of the oxide scale created during the oxidation test of the freestanding CoNiCrAlYHf coating decrease with decreasing surface roughness. Under these conditions, the oxidation behavior of this alloy primarily depends on the surface roughness, which is consistent with the aforementioned results (Figure 12). The mass change graphs show 0.6 mg·cm^−2^ for the sandblasted surface, 0.5 mg·cm^−2^ for the 100-grit sample, 0.39 mg·cm^−2^ for the 240-grit sample, 0.4 mg·cm^−2^ for the 600-grit sample, and 0.3 mg·cm^−2^ for the 2000-grit sample as a result of a rise in the surface development ratio. When comparing the roughest and smoothest samples, the sandblasted surface had nearly double the mass change value (0.6 mg·cm^−2^) of that of the 2000-grit sample (0.3 mg·cm^−2^). Therefore, oxygen from the atmosphere has a 50% larger absorption area and immediately reacts at the start of the oxidation procedure. According to the Le Chatelier–Braun direction, when the number of moles of oxygen accessible for a response increases, the number of moles of reaction products increases [34]. Furthermore, surface roughness had a significant effect on the morphology of the oxide scale. Small Al_2_O_3_ and HfO_2_ grains comprised the majority of the smooth surfaces, with the Co-/Cr-/Al-mixed oxide preferentially growing above the initial crevices of the base material. Al_2_O_3_ appeared disproportionately on the rough surfaces. The protrusions were speculated to be the locations where Al depletion rapidly occurred before HfO_2_ diffused outward through the Al_2_O_3_ grain boundaries. The interfacial decohesion was speculated to occur, owing to the strong roughening of the freestanding CoNiCrAlYHf coating surface due to sandblasting in combination with growth stresses on the thermally grown Al_2_O_3_ scale. The oxidation kinetics increased with the mass-change curve. In the early stage of oxidation, outer (Co, Ni)O and spinel (CoCr_2_O_4_) were mostly formed on the surfaces of sandblasted samples with Ra = 7.572 µm and 2000-grit samples with Ra = 0.130 µm due to the outward diffusion of metal cations reacting with oxygen at the metal/gas interface. Consequently, the outward diffusion of Cr and Al cations on the surfaces of 100-grit samples with Ra = 0.983 µm, 240-grit samples with Ra = 0.733 µm, and 600-grit samples with Ra = 0.245 µm primarily formed the outer spinel (CoCr_2_O_4_) and transitory θ-Al_2_O_3_. Due to the presence of Cr and the high temperature oxidation, the transitory Al_2_O_3_ film could be swiftly transformed into a stable single α-Al_2_O_3_ film [2,35]. The sample with the rougher surface exhibited flaws in the area close to the surface. The existence of flaws was a factor that impacted the oxidation behavior of the free-standing CoNiCrAlYHf coating. Specifically, the creation of a protective oxide scale is caused by a more significant defect concentration in the material region close to the surface. We hypothesized that the flaws in Ni would increase the coefficient of diffusion of Al within the metal. However, it is still unclear whether the imperfection acts as a manageable path for diffusion or lowers the activation energy needed for recrystallization, which leads to a higher concentration of grain boundaries. Regardless of the precise mechanism, the presence of faults triggers the production of increased amounts of protective oxide scales.

## 5. Conclusions

The surface roughness effects on the oxidation behavior of a free-standing CoNiCrAlYHf coating at 1050 °C were investigated. The major findings of this study are as follows:

1. The 2000 grit sample indicates a weight change of approximately 0.3 mg·cm^−2^, while the sandblasted sample shows a change of 0.6 mg·cm². This represents almost a two-fold decrease in mass change per unit area, indicating that reducing surface roughness can improve the oxidation resistance of free-standing CoNiCrAlYHf coating.
2. The samples with a surface roughness of 0.130 μm and polished with 2000 grits demonstrated the highest oxidation resistance because of their small exposed surface areas and thin work-hardening layers. The β-phase on the surface with Ra = 130 µm demonstrated faster growth of Al_2_O_3_ compared to the γ-phase.
3. The identical surface preparation process that resulted in a rough surface caused the formation of a thick oxide layer in the near-surface area of the material. A higher surface roughness led to the formation of a more protective oxide scale. In contrast, the surface with a smoother roughness exhibited a thin oxide scale; the interior oxide was closer to the surface. A slight reduction in surface roughness resulted in a shift in the oxidation behavior of the examined free-standing CoNiCrAlYHf coating.
4. A simple mechanical surface preparation method demonstrated that the free-standing CoNiCrAlYHf coating could move from the alumina formation region to the cobalt-chromium formation region. Polishing improved the resistance of the investigated materials to oxidation at high temperatures.

## Figures and Tables

**Table 1:** Chemical composition of the as-received investigated alloy (wt.%).

Element (wt.%)	Ni	Co	Cr	Al	Y	Hf
Sample 1	28	37.1	24	10	0.6	0.3

## Data Availability

Not applicable.
